# Supplementary figures and images for: Cysteamine Suppresses Cancer Cell Invasion and Migration in Glioblastoma through Inhibition of Matrix Metalloproteinase Activity
Source: Cancers (Basel). 2024 May 27;16(11):2029. doi: 10.3390/cancers16112029 (PMC11171184; doi:10.3390/cancers16112029)

Figure 2C

C

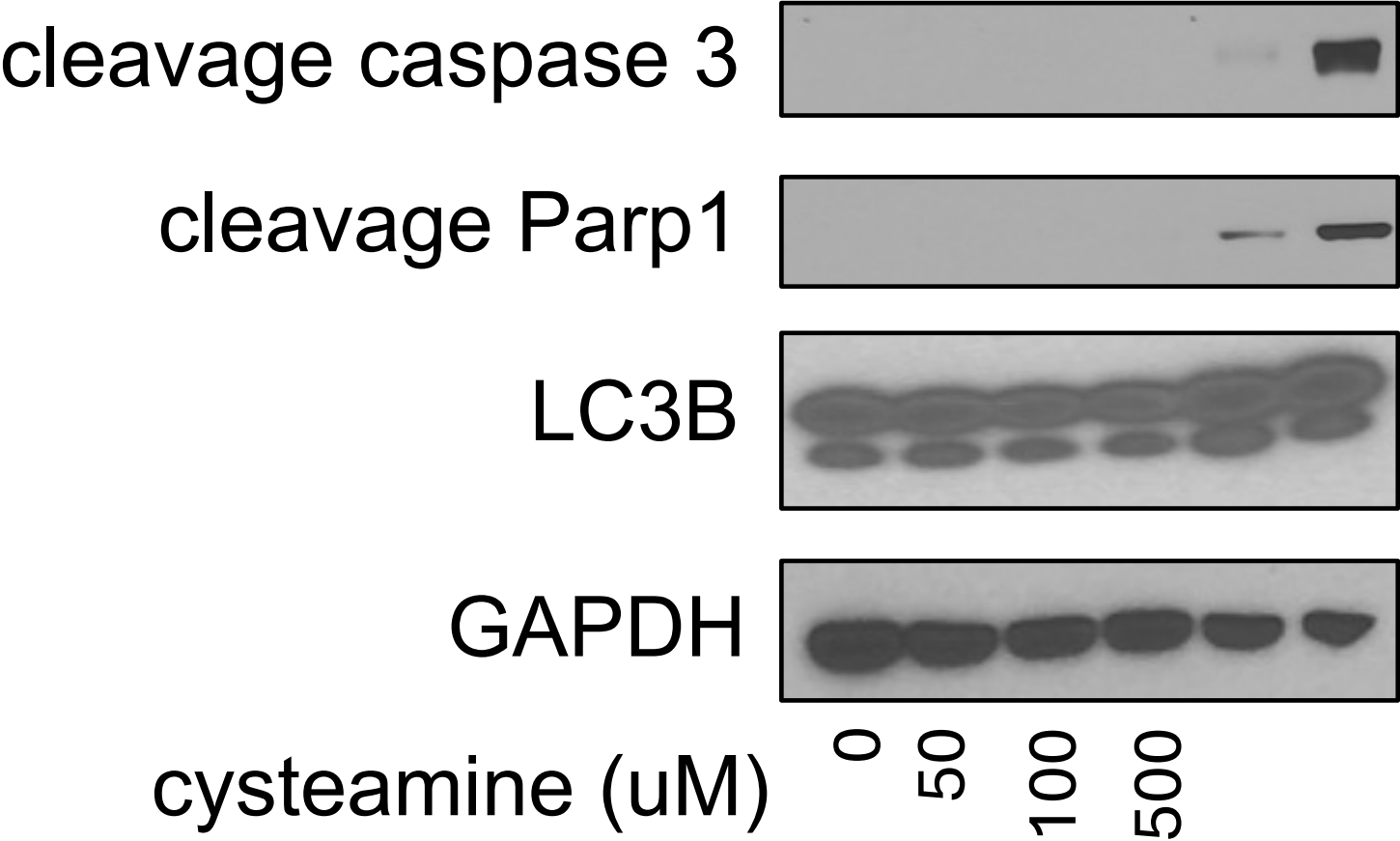

Supplement: Supplementary file 1 [file cancers-16-02029-s001.zip › cancers-2981421-File S1.pdf]

## Slide 1
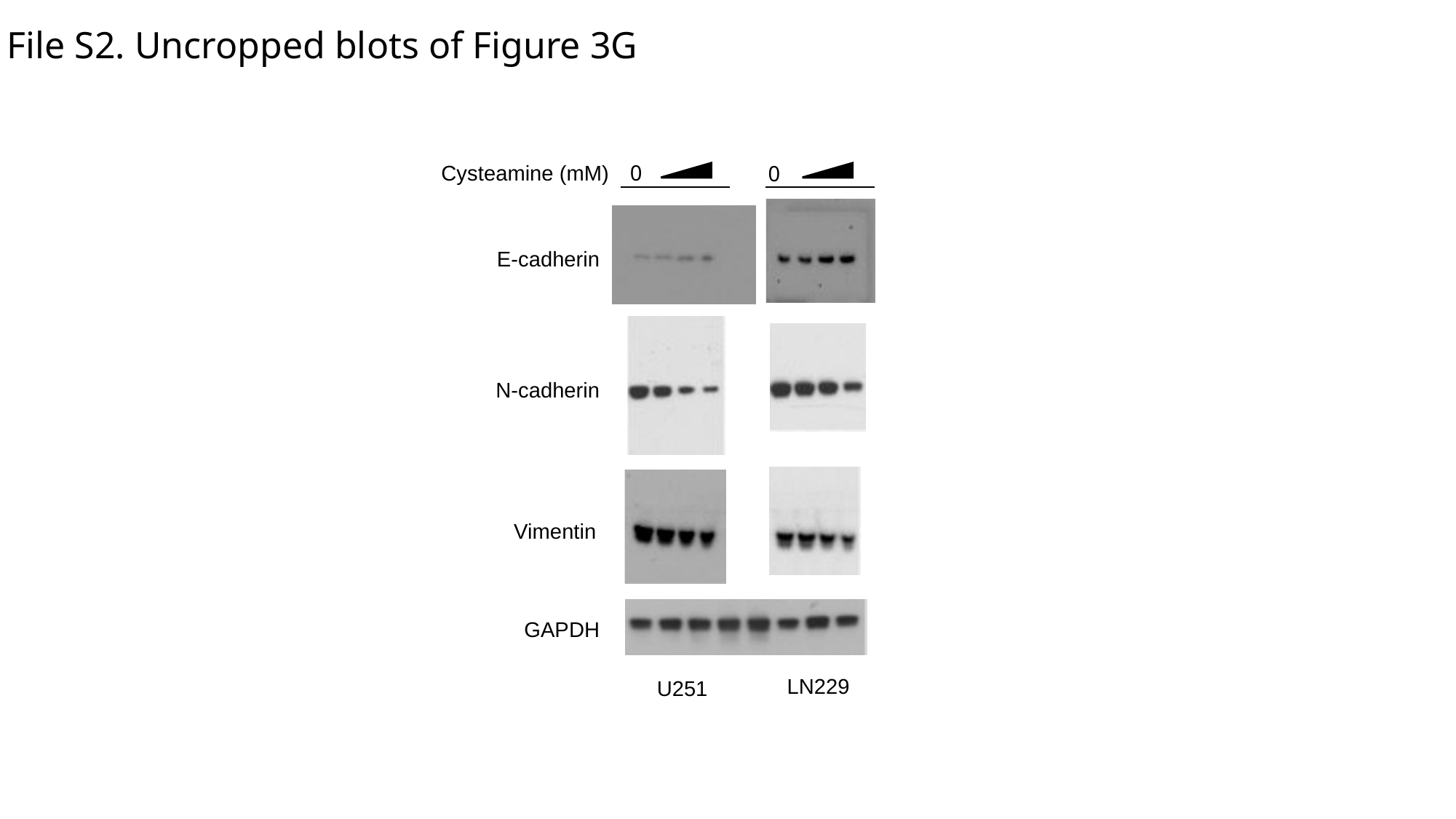

File S2. Uncropped blots of Figure 3G
0
Cysteamine (mM)
0
E-cadherin
N-cadherin
Vimentin
GAPDH
LN229
U251

Supplement: Supplementary file 1 [file cancers-16-02029-s001.zip › cancers-2981421-File S2.pptx]
